# Energy transfer of trapped electron turbulence in tokamak fusion plasmas

**DOI:** 10.1038/s41598-022-08932-4

**Published:** 2022-03-23

**Authors:** Lei Qi

**Affiliations:** Korea Institute of Fusion Energy, Daejeon, 169-148 South Korea

**Keywords:** Magnetically confined plasmas, Nuclear fusion and fission, Computational science, Nonlinear phenomena

## Abstract

The first principle gyrokinetic simulations of trapped electron turbulence in tokamak fusion plasmas demonstrate the energy transfers from the most linearly unstable modes at high $k_\theta \rho _i\sim 1$ to intermediate $k_\theta$ via parametric decay process in a short period of linear-nonlinear transition phase. Dominant nonlinear wave-wave interactions occur near the mode rational surface $m\simeq nq$. In fully nonlinear turbulence, inverse energy cascade occurs between a cutoff wave number $k_c$ and $k_\theta \rho _i\sim 1$ with a power law scaling $|\phi (k_\theta )|^2\propto k^{-3}$, while modes with $k<k_c$ are suppressed. The numerical findings show fair agreement with both the weak turbulence theory and realistic experiments on Tore Supra tokamak.

Physics of microturbulence in tokamak is a crucial subject for fusion plasmas^1^. Energy transfer of turbulent fluctuations is a fundamental and key property. Understanding the energy transfer in microturbulence can be beneficial for controlling the anomalous transport and improving the energy confinement in tokamak. Besides, the wave number spectrum is a basic physical quantity that is predictable from analytical theories and measurable in both numerical and realistic experiments. This allows us to acquire a thorough investigation of the energy transfer from analytics, simulations and experiments.

Energy transfer through nonlinear wave-wave interactions requires the conservation of energy and momentum, which can be characterized by the matching criteria of frequency $\omega _0=\omega _1+\omega _2$ and wave number $\mathbf{k}_0=\mathbf{k}_1+\mathbf{k}_2$ respectively in common triad interactions^2^. There are two distinct phenomena in nonlinear wave-wave interactions. One is parametric decay, which characterizes a three-wave interaction process involving a pump wave $(\omega _p, \mathbf{k}_p)$ with high amplitude over some threshold and two daughter wave modes $(\omega _{d1}, \mathbf{k}_{d1})$ and $(\omega _{d2}, \mathbf{k}_{d2})$. In the parametric decay process, the pump wave decays, and the two daughter wave modes grow by gaining energy from the pump wave. The three waves involved satisfy the matching conditions $\omega _p=\omega _{d1}+\omega _{d2}$ and $\mathbf{k}_p=\mathbf{k}_{d1}+\mathbf{k}_{d2}$. This process is commonly observed in various plasma waves in astrophysics^3^, solar^4^, dusty^5^, laser^6,7^ and magnetized confined plasmas^8^. The other is cascading in turbulence. Kraichnan and Kolmogorov^9^ proposed the classic energy-enstrophy cascade in 2D turbulence, which shows the power spectrum of energy intensity $E(k)\propto k^{-5/3}$ in the inverse energy cascading range and $E(k)\propto k^{-3}$ in the forward enstrophy cascading range. The Kraichnan-Kolmogorov scaling law was reproduced in the Charney–Hasegawa–Mima (CHM) 2D turbulent plasmas^10^. CHM equation can be reduced to the 2D Navier–Stokes (NS) equation in the limit of $\lambda \rightarrow 0$. Here $\lambda$ is a parameter characterize the wave number corresponding to the ratio of system size to ion Larmor radius ($\rho _i$). Equivalently, the Kraichnan-Kolmogorov scaling corresponds to the power laws of the electrostatic potential intensity $|\phi (k)|^2\propto k^{-8/3}$ for the inverse energy cascade and $|\phi (k)|^2\propto k^{-4}$ for the forward enstrophy cascade in the CHM 2D turbulence system. Here the electrostatic energy intensity *E*(*k*) connects to the electrostatic potential intensity $|\phi (k)|^2$ by $E(k)=k|\phi (k)|^2$, and the total electrostatic energy $E=\int _0^\infty E(k)dk$. This cascade picture is a result of local interactions. With disparate scale interactions it was shown $|\phi (k)|^2\propto k^{-3}/(1+k^2)^2$ in drift-wave turbulence by a simple shell model^11^.

Turbulence driven by trapped electron magnetic curvature drift resonance is primary in tokamak plasmas with a typical poloidal scale normalized to ion Larmor radius $k_\theta \rho _i\sim 1$^12^. Here $\rho _i=\sqrt{m_iT_i}/|q|B_0$ with $m_i, T_i, |q|$ being the ion mass, temperature, charge respectively, and $B_0$ is the magnetic filed. The trapped electron turbulence is in the ion scale. Understanding the energy transfer in trapped electron turbulence is of interest for tokamak plasmas, but still remains unclear. Especially, turbulence fluctuation characteristics in the short regime from late linear growth phase to early nonlinear saturation phase has not been touched yet. This transition period is therefore of particular interest. Weak turbulence theory of collisionless trapped electron (CTEM) turbulence demonstrated the cascade $|\phi (k)|^2\propto k^{-3}$ due to ion Compton scattering^13,14^. It was also shown the cascading scaling $|\phi (k)|^2\sim |\delta n(k)|^2\propto k^{-3}$ in the range $k_\theta \rho _i\sim 1$ with TEM turbulence dominant from experimental measurements on Tore Supra tokamak^15^.

In this paper, we investigate the properties of energy transfer in collisionless trapped electron turbulence. Turbulent fluctuation spectrum is found near the mode rational surface $m\simeq nq$, where dominant wave-wave interactions occur. Here *m*, *n*, *q* are poloidal, toroidal mode number and magnetic safety factor respectively. Energy transfers from high $k_\theta \rho _i\sim 1$ modes to a transitional intermediate *k* modes in a short period of linear-nonlinear transition via parametric decay process. Inverse energy cascade is eventually observed with $|\phi (k)|^2\propto k^{-3}$ starting between a finite cutoff wave number $k_c$ and $k_\theta \rho _i\sim 1$, while modes with $k<k_c$ are suppressed. The cutoff wave number $k_c$ can be defined and measured as the wave number where the spectral intensity $|\phi (k)|^2$ peaks and starts decaying monotonically to higher *k*. These findings show fairly good agreement with weak turbulence theory and experimental measurements on Tore Supra tokamak.

## Methods

### Numerical experiment platform

All simulations presented in this article were conducted on the numerical experiment platform gKPSP^16,17^. gKPSP is a global gyrokinetic particle-in-cell (PIC) code to simulate electrostatic turbulence in general tokamak configurations. In this program, ions are evolved following the gyrokinetic equations^18,19^, while kinetic trapped electrons are governed by the bounce-averaged kinetic theory^20^ with drift-kinetic passing electrons passively responding to fluctuations. A zonal flow conserving Krook operator^21^ is employed to control discrete particle noises and to provide source energy for maintaining the temperature and density profiles so that their averages in time remain close to initial values. The simulation program has been successfully applied to study CTEM turbulence in various situations^22–24^.

### Simulation setup

**Figure 1:**
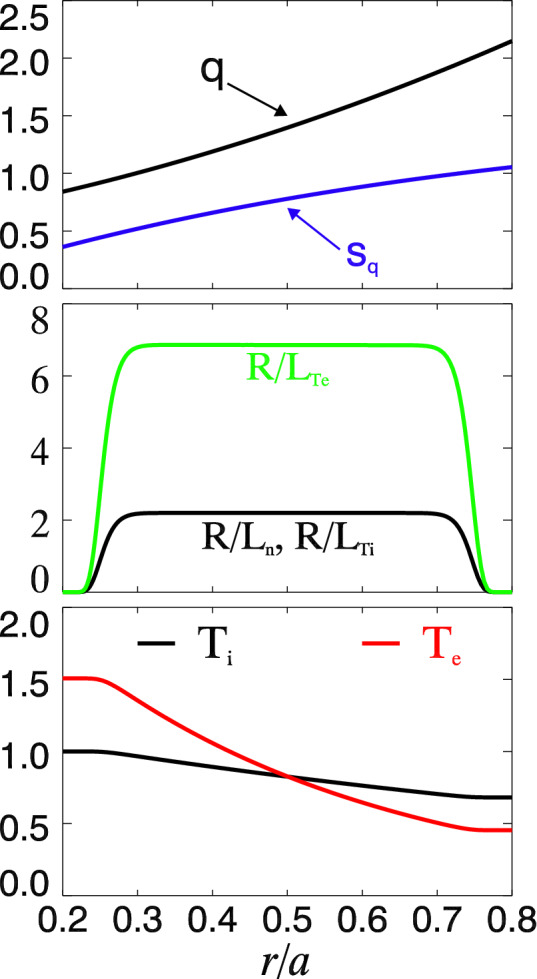
Simulation setup of radial profiles. Safety factor *q* and magnetic shear $\hat{s}_q$ (top figure), normalized gradients $R/L_n=R/L_{Ti}=2.2$ and $R/L_{Te}=6.9$ (middle figure), ion $T_i$ and electron $T_e$ temperature (bottom figure).

Electron temperature gradient $\nabla T_e$ provides the main source energy for the trapped electron turbulence in this study. The normalized electron temperature gradient $R/L_{Te}$ varies from 5 to 9, while ion temperature gradient $R/L_{Ti}=2.2$ and electron (ion) density gradient $R/L_n=2.2$ are fixed. $R=1.86m$ is the tokamak major radius and $L_\alpha \equiv -(d\ln \alpha /dr)^{-1}$. The minor radius is $a=0.666m$ with $a/\rho _i\simeq 220$ at middle radius, and the magnetic field $B_0=1.91T$. Hydrogen plasma is used in the simulation with mass ratio $m_i/m_e=1836$. Radial profiles of the safety factor *q*, magnetic shear $\hat{s}_q$, $R/L_n$, $R/L_{Ti}$, $R/L_{Te}=6.9$, ion (electron) temperature $T_i$ ($T_e$) are presented in Fig. 1. Finite density and temperature gradients are set up in the range $r/a\in [0.25, 0.75]$, where micro-instability and turbulence can be destabilized. The radial profiles are set up to assure at the middle radius $r=0.5a$, $q\simeq 1.4$, $\hat{s}_q=0.78$ and $T_i=T_e$. In nonlinear simulations, we set up the maximum toroidal wave number $n_{max}=116$, which corresponds to a maximum poloidal wave number $k_\theta \rho _i$ slightly larger than 1.0 at mid-radius, while modes with $n>n_{max}$ are sunk. Zonal flow ($n=0$) self-consistently driven by the CTEM turbulence is included in all nonlinear simulations. Collision operator is turned off, thus collisionless trapped electron turbulence can be destabilized with the spatial scale $k_\theta \rho _i\sim 1$.

## Results

### Parametric decay in linear-nonlinear transition

**Figure 2:**
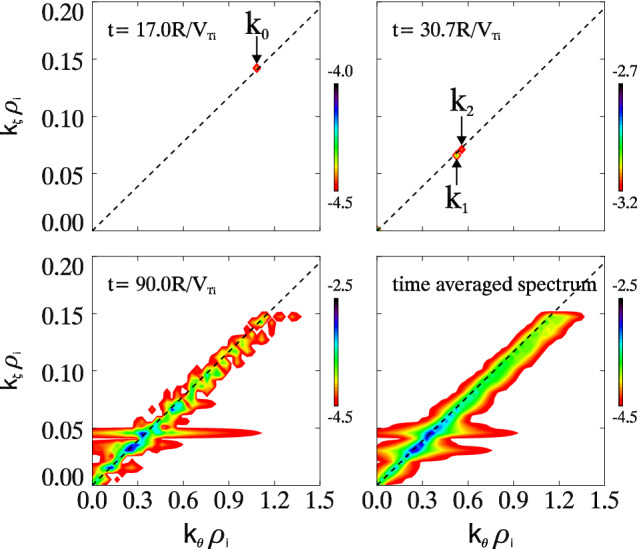
Contour plots of the turbulence electrostatic potential spectra $\log(|\phi (k_\theta , k_\zeta )|)$ in the poloidal-toroidal wave number ($k_\theta \rho _i-k_\zeta \rho _i$) domain. At time $t=17.0R/V_{Ti}$ (late linear phase), $30.7R/V_{Ti}$ (early nonlinear phase), $90.0R/V_{Ti}$ (fully nonlinear phase) and time-averaged spectrum in the nonlinear saturation. Black dash lines indicate the approximate mode rational surface.

**Figure 3:**
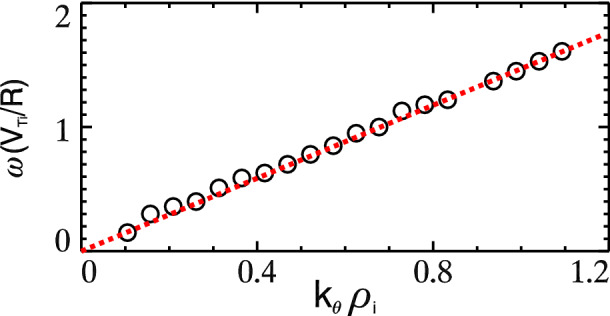
Frequency (depicted as circles) of the most linearly unstable modes as a function of poloidal wave number $k_\theta \rho _i$. Red dash line shows the approximate linear relation $\omega \propto k_\theta$.

The spectra of electrostatic potential fluctuation $\phi (t, k_\theta , k_\zeta )$ is measured at the middle radius $r/a=0.5$ in the numerical experiment of the case with $R/L_{Te}=6.9$. Three typical time regimes are selected, the late linear phase at time $t=17.0R/V_{Ti}$ (Here $V_{Ti}=\sqrt{T_i/m_i}$ is the ion thermal velocity.), the early nonlinear saturation $t=30.7R/V_{Ti}$ and the fully nonlinear phase $t=90.0R/V_{Ti}$. Contours of $\log(|\phi |)$ in poloidal-toroidal wave number $(k_\theta , k_\zeta )$ domain are plotted for the three selected time regimes as well as the time-average one in the nonlinear stage in Fig. 2. The most linearly growing mode can be found at $t=17.0R/V_{Ti}$ as ${\mathbf{k}_0}\equiv (k_{\theta 0}\rho _i, k_{\zeta 0}\rho _i)\simeq (1.08, 0.142)$. This mode decays right away entering the nonlinear stage, and the two most dominant modes can be recognized at $t=30.7R/V_{Ti}$ as $\mathbf{k}_1\equiv (k_{\theta 1}\rho _i, k_{\zeta 1}\rho _i)\simeq (0.525, 0.066)$ and $\mathbf{k}_2\equiv (k_{\theta 2}\rho _i, k_{\zeta 2}\rho _i)\simeq (0.558, 0.071)$. These three modes can match the parametric decay criteria $\mathbf{k}_0\simeq \mathbf{k}_1+\mathbf{k}_2$. Frequency of the most linearly unstable modes is shown by circles in Fig. 3 as a function of poloidal wave number $k_\theta \rho _i$. The poloidal mode number is calculated by $k_\theta =nq/r$, and the frequency is measured at different *n* values. Red dashed line in the figure indicates the linear relation between frequency and wave number, i.e., $\omega \propto k_\theta$. Therefore, for three wave modes matching the wave number condition $k_{\theta 0}\simeq k_{\theta 1}+k_{\theta 2}$, the frequency criteria of triad interactions can be matched up consistently $\omega _0\simeq \omega _1+\omega _2$.

**Figure 4:**
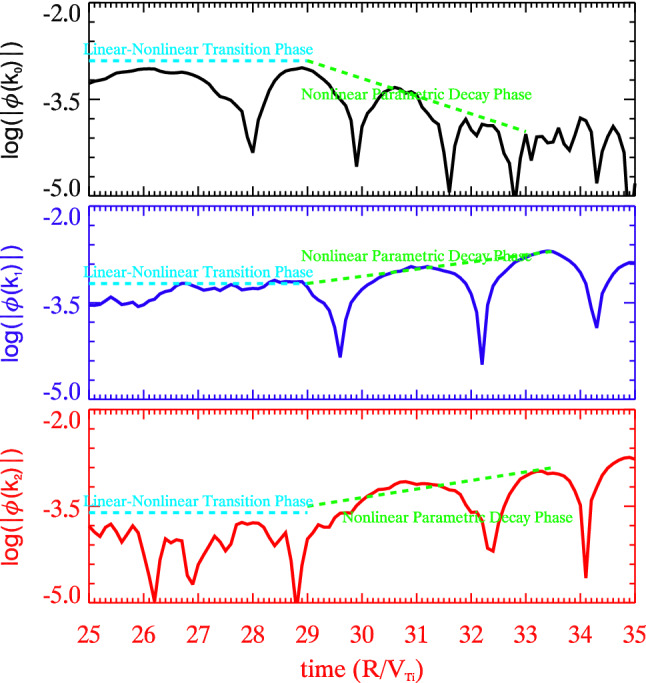
Parametric decay process. Time evolutions of $\log |\phi |$ in the linear-nonlinear transition phase for the most linearly growing mode $\mathbf{k}_0$ (top-black), the two daughter wave modes $\mathbf{k}_1$ (middle-blue) and $\mathbf{k}_2$ (bottom-red).

The parametric decay process in the linear-nonlinear transition phase can be clearly observed from the time evolution plots of $\log (|\phi (\mathbf{k})|)$ for the three modes $\mathbf{k}_0$ (top), $\mathbf{k}_1$ (middle) and $\mathbf{k}_2$ (bottom), as shown in Fig. 4. After the linear growth, wave modes enter the linear-nonlinear transition phase, where waves cease growing and saturate. This phase is very short $\sim 1$ period, and the system starts the nonlinear evolution. The most linearly unstable mode $\mathbf{k}_0$ decays within a couple of periods and transfers energy to two daughter wave modes $\mathbf{k}_1$ and $\mathbf{k}_2$ by the matching criteria. The daughter modes absorb the energy and grow in the mean time. The parametric decay process is the dominant energy transfer mechanism in the early trapped electron turbulence.

Since in the tokamak configuration, poloidal wave number $k_\parallel \propto m-nq$, $k_\parallel \simeq 0$ around the mode rational surface $m-nq\simeq 0$. Therefore, from the theoretical point of view, the Landau damping becomes weaker as $k_\parallel$ approaches 0, which has also been demonstrated in the experiment^25^ showing that large $k_\parallel$ modes are Landau damped. Due to the minimum Landau damping, the most unstable modes locate around the rational surface $m\simeq nq$, which indicates the surface $k_\zeta \simeq \alpha k_\theta$ ($\alpha$ a constant). This is also demonstrated from the simulations and shown by the black dashed lines in Fig. 2. Other gyrokinetic codes such as GT5D and ORB5 demonstrated that keeping $2\Delta m+1=11$ poloidal modes per toroidal mode and per magnetic surface is sufficient to converge the ion heat diffusivity and the turbulence spectra from ion temperature gradient (ITG) driven turbulence^26^, which is consistent with the aforementioned findings for TEM. Here $|m-nq|<\Delta m$ defines the parameter $\Delta m$. Since the linear frequency of trapped electron instability $\omega \propto k_\theta$, the triad wave-wave interactions can automatically occur on the mode rational surface. Therefore, the energy is distributed around the rational surface, even in the nonlinear turbulence as shown by the $t=90.0R/V_{Ti}$ and time-average spectra in Fig. 2. Poloidal spectrum broadening can be observed at some high amplitude $k_\zeta$ modes in the figure.

**Figure 5:**
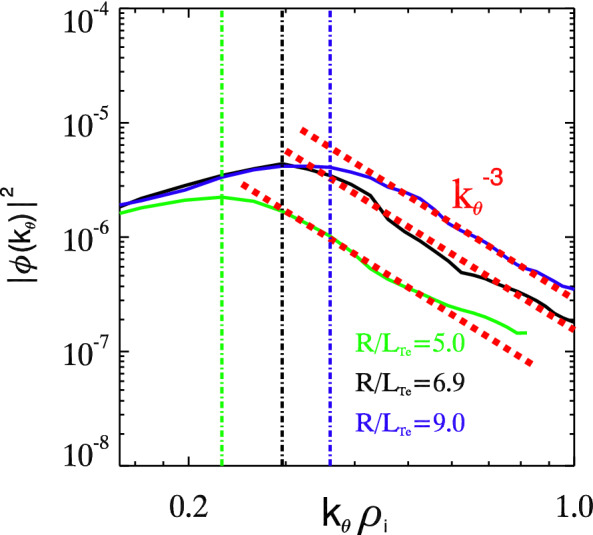
Poloidal wave number spectra of $|\phi (k_\theta )|^2$ for three cases $R/L_{Te}=5.0$ (green), 6.9 (black) and 9.0 (blue) in log-log scale. Red dash lines indicate the scaling of inverse energy cascade $|\phi (k_\theta )|^2\propto k_\theta ^{-3}$. Vertical dash-dot lines depict the cutoff wave numbers for cases with the same color.

### Inverse energy cascade scaling law

Inverse energy cascade $|\phi (k_\theta )|^2\propto k_\theta ^{-3}$ can be observed in the poloidal wave number spectrum in the regime $k_\theta \rho _i\sim 1$ as shown in Fig. 5. For the sake of generality, we present three cases with different electron temperature gradients $R/L_{Te}=5.0$ (green), 6.9 (black) and 9.0 (blue). The spectra are obtained by time averaging in the saturation stage from $t=40R/V_{Ti}$ up to $t=300R/V_{Ti}$, when the turbulence has been fully evolved. The power law scaling $k_\theta ^{-3}$ fits well for all cases, as indicated by red dash lines. This cascade scaling from the simulations agrees well with the weak turbulence kinetic theory of trapped electron drift wave turbulence, e.g. Eq. (81) in Ref.^13^ and Eq. (27) in Ref.^14^. The kinetic theories predict the inverse energy cascading $|\phi (k_\theta )|^2\propto k_\theta ^{-3}$ due to ion Compton scattering. This power law scaling factor $-3$ is close to the classic Kolmogorov inverse energy cascade scaling factor $-8/3$. On the other hand, the shell model^11^ of drift wave turbulence predicts the inverse energy cascade scaling $|\phi (k)|^2\propto k^{-3}/(1+k^2)^2$, which is also close to our simulation results $|\phi (k_\theta )|^2\propto k_\theta ^{-3}$ in trapped electron turbulence for $k_\theta \rho _i <1$.

Measurements on Tore Supra tokamak show the spectrum power law scaling of density fluctuation $|\delta n(k)|^2\propto k^{-3\pm 0.5}$ in the length scale regime $k\rho _i\sim 1$^15^. The analysis of the experimental parameters also show the dominance of trapped electron drift wave instability. Therefore, the cascade $|\phi (k_\theta )|^2\propto k_\theta ^{-3}$ from our simulations of trapped electron turbulence agrees well with the experimental measurements on Tore Supra tokamak. We have so far demonstrated consistent inverse energy cascade $|\phi (k_\theta )|^2\propto k_\theta ^{-3}$ of trapped electron turbulence with $k_\theta \rho _i\sim 1$ from gyrokinetic simulations, weak turbulence theories and experiments.

One can also observe from Fig. 5 that the inverse energy cascade starts at a cutoff wave number $k_c$, while spectra in the range $k_\theta <k_c$ are suppressed. Nonlinear interactions between disparate $k_\theta$ scales via trapped electron scattering, which is a resonance process between the beat wave and the trapped electron precession drift frequencies, is believed to be the mechanism suppressing modes with $k_\theta <k_c$^14^. Vertical dash-dot lines depict approximately the cutoff wave number $k_c\rho _i\sim 0.2-0.4$ for the three cases with the same color in Fig. 5. The cutoff wave number increases with electron temperature gradient $R/L_{Te}$. This finding agrees fairly with the prediction from weak turbulence theory. Eq. (33) in Ref.^14^ gives the theoretical expression of the cutoff wave number $k_c$ ($k_L$ in the equation). We can obtain the dependence of the cutoff wave number $k_c$ on $\eta _e\equiv L_n/L_{Te}$ by evaluating the derivative $dk_c/d\eta _e>0$ from the Eq. (33) in Ref.^14^. It is straight forward to yield $dk_c/d\eta _e>0$ if $R/GL_n-3/2>0$. $G\simeq 0.64\hat{s}+0.57=1.07$ by averaging over the azimuthal angle with the magnetic shear $\hat{s}=0.78$ in the simulations, and $R/L_n=2.2$. So with simulation parameters, we have the cutoff wave number increases monotonically with $R/L_{Te}$. The simulation results are consistent with the theoretical predictions.

On the other hand, the experiments on Tore Supra tokamak also demonstrate the cutoff wave number, where the inverse energy cascade $|\delta n(k)|^2\propto k^{-3\pm 0.5}$ starts, e.g., Figures 2,3, 5–7 in Ref.^15^. The cutoff wave number $k_c\rho _i\sim 0.3-0.5$ is close to our simulation observations considering the difference of parameters. The spectra with $k<k_c$ is suppressed to be near a constant from the experimental measurements.

From simulations, theories and experiments, we show consistent feature of cutoff wave number in the turbulence spectra. Modes with $k<k_c$ is suppressed via trapped electron scattering, while spectra in the regime $k>k_c$ to $k\rho _i\sim 1$ decay monotonically with a cascading scaling $k^{-3}$. We note that this characteristic supports the trapped electron turbulence as a potential candidate for the quasi-coherent mode (QCM) observed in experiments^27^. The trend of the cutoff wave number $k_c$ on $\eta _e$ observed from the simulation as well as the weak turbulence theory predictions (eg. Eq. (33) in Ref.^14^) can be inspiring for the experimental study of trapped electron turbulence in tokamaks.

**Figure 6:**
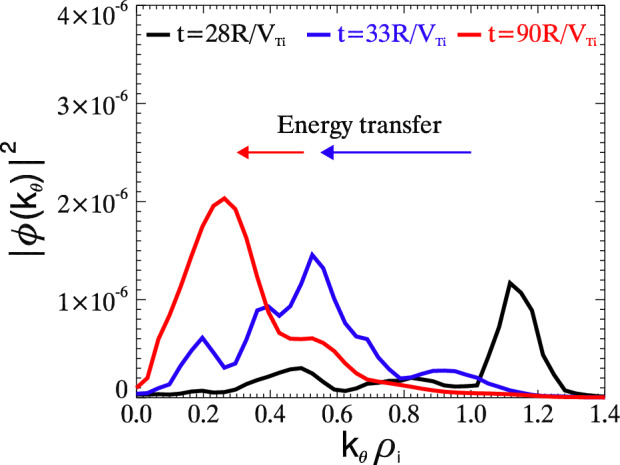
Overall picture of the energy transfer process. Plot of $|\phi (k_\theta )|^2$ for three different time $t=28R/V_{Ti}$ (black), $33R/V_{Ti}$ (blue) and $90R/V_{Ti}$ (red) shows the energy transfer in trapped electron turbulence.

## Discussions

Figure 6 presents the overall picture of the energy transfer in the trapped electron turbulence. Near the end of the linear phase $t=28R/V_{Ti}$, the most growing mode locates at high $k_\theta \sim 1$. The energy transfers from the most linearly unstable mode to intermediate $k_\theta$ modes via parametric decay process within a short period of linear-nonlinear transition phase. The blue line depicts the spectrum at $t=33R/V_{Ti}$ after the parametric decay. The turbulence eventually evolves to an inverse cascade $|\phi (k_\theta )|^2\propto k_\theta ^{-3}$ between the cutoff wave number $k_c$ and $k_\theta \rho _i\sim 1$ due to ion Compton scattering. Modes with $k<k_c$ are suppressed by disparate nonlinear interactions via trapped electron scattering. Dominant nonlinear wave-wave interactions occur near the mode rational surface $m\simeq nq$. Findings from simulations agree well with both analytical weak turbulence theory and realistic experiments on Tore Supra Tokamak.

For the future work, characteristics of energy transfer in ITG turbulence and ITG-TEM co-existence turbulence comparing to CTEM turbulence draw us the further interest. We also note that it has been extensively studied in experiments^28,29^ showing that the inverse energy cascade has strong effects on the generation of zonal flow and therefore the evolution and suppression of plasma turbulence. The findings inspire us to study the impacts of energy transfer on the zonal flow generation in micro-turbulence with our numerical experimental platform in future research.

## Data Availability

The data is available from the corresponding author upon reasonable request.

## References

[1] Horton W (1999). Drift waves and transport. Rev. Mod. Phys..

[2] Tsytovich V (1970). Nonlinear Effects in Plasma.

[3] Chen L, Zonca F (2011). Gyrokinetic theory of parametric decays of kinetic Alfvén waves. EPL.

[4] Shoda M, Yokiyama T, Suzuki TK (2018). Frequency-dependent Alfvén-wave propagation in the solar wind: Onset and suppression of parametric decay instability. ApJ.

[5] Jamil M (2011). The parametric decay of dust ion acoustic waves in non-uniform quantum dusty magnetoplasmas. Phys. Plasmas.

[6] Rosenbluth MN, Liu CS (1972). Excitation of plasma waves by two laser beams. Phys. Rev. Lett..

[7] Grebogi C, Liu CS (1980). Parametric decay of extraordinary electromagnetic waves into two upper hybrid plasmons. J. Plasma Phys..

[8] Baek SG (2013). Measurements of ion cyclotron parametric decay of lower hybrid waves at the high-field side of Alcator C-Mod. Plasma Phys. Control. Fusion.

[9] Kraichnan RH (1967). Inertial ranges in two-dimensional turbulence. Phys. Fluids.

[10] Watanabe T, Fujisaka H (1997). Dynamical scaling law in the development of drift wave turbulence. Phys. Rev. E.

[11] Gürcan ÖD (2009). Wave-number spectrum of drift-wave turbulence. Phys. Rev. Lett..

[12] Adam JC, Tang WM, Rutherford PH (1976). Destabilization of the trapped-electron mode by magnetic curvature drift resonances. Phys. Fluids.

[13] Gang FY, Diamond PH, Rosenbluth MN (1991). A kinetic theory of trapped-electron-driven drift wave turbulence in a sheared magnetic field. Phys. Fluids B Plasma Phys..

[14] Hahm TS, Tang WM (1991). Weak turbulence theory of collisionless trapped electron driven drift instability in tokamaks. Phys. Fluids B Plasma Phys..

[15] Hennequin P (2004). Scaling laws of density fluctuations at high-k on Tore Supra. Plasma Phys. Control. Fusion.

[16] Qi L, Kwon JM, Hahm TS, Jo G (2016). Gyrokinetic simulations of electrostatic microinstabilities with bounce-averaged kinetic electrons for shaped tokamak plasmas. Phys. Plasmas.

[17] Kwon JM, Qi L, Yi S, Hahm TS (2017). ITG-TEM turbulence simulation with bounce-averaged kinetic electrons in tokamak geometry. Comput. Phys. Commun..

[18] Hahm TS (1988). Nonlinear gyrokinetic equations for tokamak microturbulence. Phys. Fluids.

[19] Brizard A (1989). Nonlinear gyrokinetic Maxwell–Vlasov equations using magnetic coordinates. J. Plasma Phys..

[20] Fong BH, Hahm TS (1999). Bounce-averaged kinetic equations and neoclassical polarization density. Phys. Plasmas.

[21] McMillan BF (2008). Long global gyrokinetic simulations: Source terms and particle noise control. Phys. Plasmas.

[22] Qi L, Kwon JM, Hahm TS, Yi S (2017). Bounce-averaged gyrokinetic simulation of trapped electron turbulence in elongated tokamak plasmas. Nucl. Fusion.

[23] Qi L, Kwon JM, Jhang H, Hahm TS, Leconte M (2020). Nonlinear gyrokinetic analysis of linear ohmic confinement to saturated ohmic confinement transition. Nucl. Fusion.

[24] Qi L, Choi MJ, Kwon JM, Hahm TS (2020). Role of zonal flow staircase in electron heat avalanches in KSTAR L-mode plasmas. Nucl. Fusion.

[25] Zweben SJ, Medley SS (1989). Visible imaging of edge fluctuations in the TFTR tokamak. Phys. Fluids B.

[26] Jolliet S (2012). Parallel filtering in global gyrokinetic simulations. J. Comput. Phys..

[27] Lee W (2021). Study of the origin of quasi-coherent modes in low-density KSTAR ECH plasmas. Nucl. Fusion.

[28] Xia H, Shats MG (2004). Spectral energy transfer and generation of turbulent structures in toroidal plasma. Phys. Plasmas.

[29] Shen Y (2019). The scale-to-scale energy transfers correlated with the development of turbulence in toroidal plasmas. Nucl. Fusion.

